# Lgr5 in cancer biology: functional identification of Lgr5 in cancer progression and potential opportunities for novel therapy

**DOI:** 10.1186/s13287-019-1288-8

**Published:** 2019-07-29

**Authors:** Liangliang Xu, Weiping Lin, Longping Wen, Gang Li

**Affiliations:** 1 Key Laboratory of Orthopaedics and Traumatology, The First Affiliated Hospital of Guangzhou University of Chinese Medicine, Guangzhou University of Chinese Medicine, Guangzhou, People’s Republic of China; 2 Laboratory of Orthopaedics and Traumatology, Lingnan Medical Research Center, Guangzhou University of Chinese Medicine, Guangzhou, People’s Republic of China; 3 Department of Orthopaedics and Traumatology, Faculty of Medicine, Prince of Wales Hospital, The Chinese University of Hong Kong, Shatin, Hong Kong SAR PRC; 4 Stem Cells and Regenerative Medicine Laboratory, Lui Che Woo Institute of Innovative Medicine, Li Ka Shing Institute of Health Sciences, The Chinese University of Hong Kong, Prince of Wales Hospital, Shatin, Hong Kong SAR PRC; 5 Nanobio Laboratory, Institute of Life Sciences, South China University of Technology, Guangzhou, Guangdong People’s Republic of China; 6 The CUHK-ACC Space Medicine Centre on Health Maintenance of Musculoskeletal System, The Chinese University of Hong Kong Shenzhen Research Institute, Shenzhen, People’s Republic of China

**Keywords:** Lgr5, Wnt/β-catenin signaling, Cancer, Metastasis

## Abstract

Cancer remains one of the leading lethal diseases worldwide. Identifying biomarkers of cancers might provide insights into the strategies for the development of novel targeted anti-cancer therapies. Leucine-rich repeat-containing G protein-coupled receptor 5 (Lgr5) has been recently discovered as a candidate marker of cancer stem cell populations. Aberrant increased expression of Lgr5 may represent one of the most common molecular alterations in some human cancers, leading to long-term potentiation of canonical Wnt/β-catenin signaling. On the other hand, however, Lgr5-mediated suppression in canonical Wnt/β-catenin signaling has also been reported in certain cancers, such as B cell malignancies. Until now, therapeutic approaches targeting Lgr5-associated signaling axis are not yet clinically available. Increasing evidence have indicated that endogenous Lgr5^+^ cell population is implicated in tumor initiation, progression, and metastasis. This review is to summarize our current knowledge about the importance of Lgr5 in cancer biology and the underlying molecular mechanisms of Lgr5-mediated tumor-promoting/suppressive activities, as well as potentially useful preventive strategies in treating tumor. Therefore, targeted therapeutic modulation of Lgr5^+^ cancer cell population by targeting Wnt/β-catenin signaling through targeted drug delivery system or targeted genome editing might be promising for potential novel anti-cancer treatments. Simultaneously, combination of therapeutics targeting both Lgr5^+^ and Lgr5^−^ cancer cells may deserve further consideration considering the plasticity of cancer cells. Also, a more specific targeting of cancer cells using double biomarkers may be much safer and more effective for anti-cancer therapy.

## Introduction

Cancer remains among the most challenging diseases worldwide although extensive studies have been performed and novel systemic treatment advances during recent decades. In general, most cancer deaths are attributed to tumor recurrence and metastasis [1, 2]. Therefore, it is necessary and imperative to better understand the cellular and molecular mechanisms underlying cancer recurrence and tumor metastasis.

Increasing evidence suggest that both intrinsic properties of cancer cells and host organ microenvironment participate actively in tumor metastasis [3]. The patterns of organ-specific metastasis of tumor are determined by the concept of “seed (the cancer cell) and soil (the secondary organ),” which was first proposed by Dr. Stephen Paget in 1889 [4, 5]. Hence, the process of tumor progression has been regarded as a result of an evolving crosstalk between different cell types within the tumor and its surrounding host tissues and organs or tumor stroma [6–8]. It has been well documented that tumor sites are in a hypoxic and inflammatory microenvironment with the release of various chemokines, cytokines, and growth factors that recruit bone marrow-derived cells (e.g., mesenchymal stem cells, macrophages) into the tumor sites via interactions with the surface receptors of bone marrow-derived cells [9–11]. These factors secreted by tumor cells include interleukins, tumor necrosis factor-α (TNF-α), stromal cell-derived factor 1 (SDF-1), and other identified and unidentified inflammatory transmitters [12–15]. Also, the tumor microenvironment contains various inflammatory cells, including myeloid cell subpopulations, innate and adaptive immune cells NK(T) cells, and B and T cells [16, 17]. Thus, this dynamic tumor microenvironment with hypoxia and inflammation may be responsible for tumor cell variants through genomic instability, genomic heterogeneity, and epigenetic alterations, making tumor unpredictably behavioral diversified and difficult to cure [18, 19].

It is generally believed that chronic inflammatory diseases are well-recognized causes of cancer, accounting for 20% of all cancer deaths worldwide [20–22]. A recent study reported that the development of a tumor-promoting immune environment in chronic inflammation was initiated through a regulatory T cell-dependent mechanism. Thus, interleukin-33 (IL-33)/regulatory T cells (Tregs) axis may become a potential therapeutic target for the treatment of chronic inflammation-associated cancers [21].

Tumor recurrence is also a great challenge for cancer therapy besides tumor invasion [23, 24], which may be attributed to abnormal immune response. Usually, a metastatic disease occurs after a prolonged period of dormancy, when disseminated cancer is present but clinically undetectable. It has been demonstrated that neutrophils recruited during lung inflammation could initiate the awakening of dormant cancer cells [25]. A recent study further discovered that neutrophil extracellular traps formed by neutrophils during lipopolysaccharide- or tobacco smoke-induced lung inflammation are required to awaken dormant cancer cells, causing tumor metastasis in mice consequently [26]. Therefore, the process of tumor progression behaves very much like an immune disease to some extent [27].

## Wnt signaling, Lgr5, and cancer

Wnt signaling pathway has a crucial role in embryonic development, tissue regeneration, and diseases, in particular cancer [28–30]. The canonical Wnt/β-catenin signaling is essential for regulating the stemness, proliferation, and differentiation of several adult stem cell niches, such as hair follicles in the skin, the intestinal crypt, the mammary gland, and the hematopoietic tissues [31–36]. In the meanwhile, however, canonical Wnt signaling is also implicated in the formation and progression of various human cancers [30, 37–39].

**Fig. 1:**
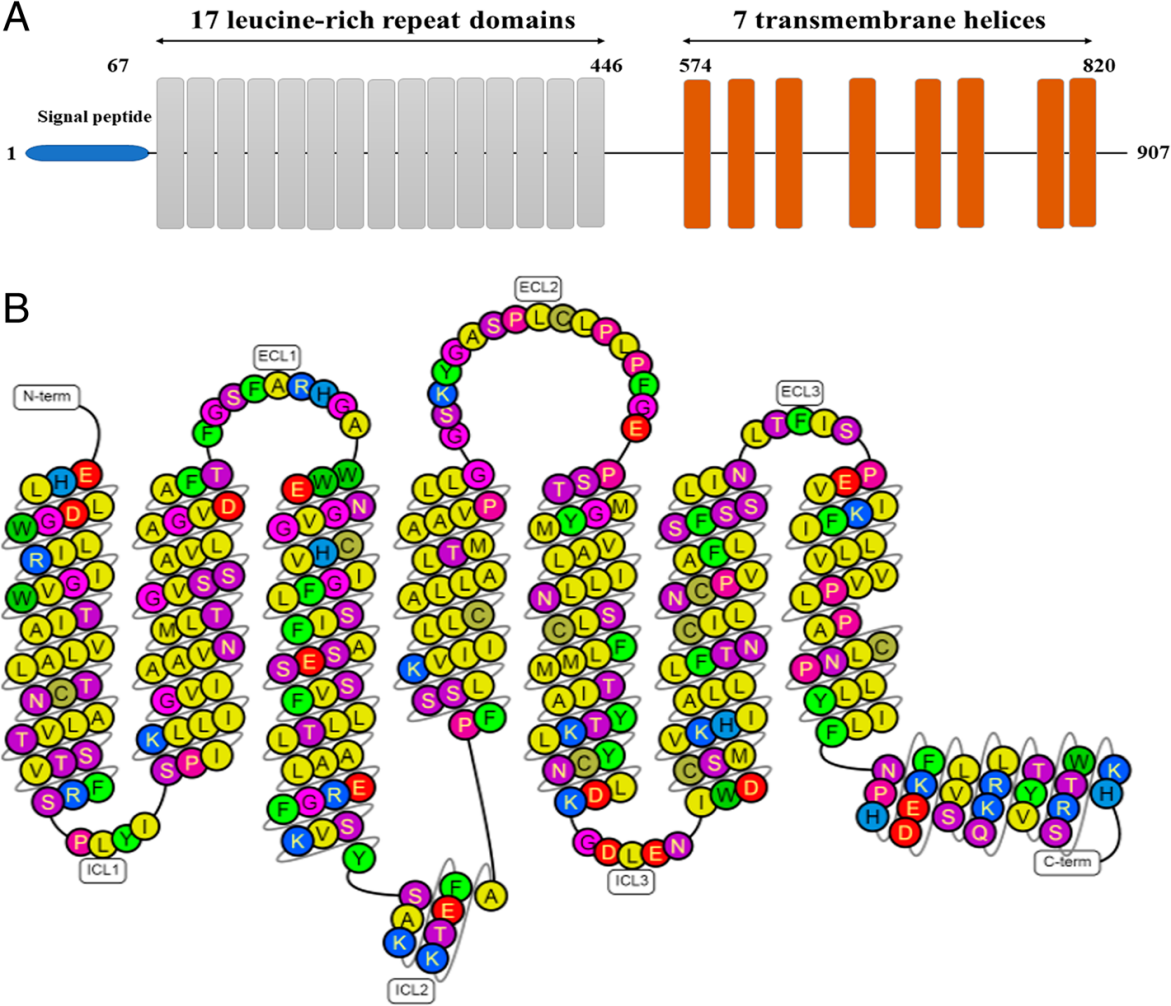
The schematic illustration of the general structure of Lgr5. **a** Lgr5 comprises of a signal peptide (blue) followed by 17 leucine-rich repeat (LRR) domains (gray). Also, it contains a linker region between the last LRR and the first transmembrane (TM) domain, followed by a seven helical TM domain homologs to rhodopsin-like G protein receptors (GPCRs). **b** The diagram showing the structure of human Lgr5 is produced by GPCRdb (http://docs.gpcrdb.org/index.html). ICL, intracellular loops; ECL, extracellular loops

Leucine-rich repeat-containing G protein-coupled receptor 5 (Lgr5), also known as G protein-coupled receptor 49 (GPR49), is an “orphan” receptor belonging to the G protein-coupled receptor (GPCR) family [40, 41]. Lgr5 was originally discovered and cloned by Dr. Aaron J. W. Hsueh’s group in 1998 [42]. The *Lgr5* gene is ~ 144 kb long and is located on chromosome 12 at position 12q22–q23. And its protein structure has been presented in Fig. 1 [43]. Accumulating body of evidence have indicated that Lgr5 is essential for normal embryonic development and emerges as a novel bona fide marker of adult stem cells in various organs and tissues exhibiting multi-biologic functions [34, 44–54].

Notably, Lgr5 has been demonstrated upregulated in various cancer tissues, such as basal cell carcinomas, hepatocellular carcinomas, colorectal tumors, and ovarian tumors [55, 56]. In general, Lgr5 modulates canonical Wnt signaling strength through binding to its ligand R-spondin [41, 57]. Lgr5 potentiates Wnt/β-catenin signaling pathway, thereby stimulating cancer stem cell proliferation and self-renewal [58, 59]. Lgr5 has been demonstrated to promote cancer cell mobility, tumor formation, and epithelial-mesenchymal transition in breast cancer cells via activation of Wnt/β-catenin signaling. Notably, Lgr5 is required for the maintenance of breast cancer stem cells [58]. Furthermore, positive correlations between high expression of Lgr5 and shorter survival of patients have been reported [2]. Studies have further demonstrated that Lgr5 regulates the malignant phenotype in a subset of patient-derived glioblastoma stem cells, which may represent as a potential predictive marker of glioblastoma [60]. On the other hand, however, Lgr5 have been shown to negatively regulate Wnt/β-catenin signaling in some special occasions [61]. Importantly, numerous studies using genetic lineage tracing analysis or detection by antibodies against Lgr5 have indicated Lgr5 as biomarkers of cancer stem cells of various human cancer types, such as adenocarcinoma, glioblastoma, and colorectal and breast cancers [62–71].

Interestingly, canonical and non-canonical Wnt signaling pathways seem to exhibit opposing effects on tumor growth [72–75]. The canonical Wnt signaling stimulates liver growth and regeneration [76], and is reported to be activated in “well-differentiated” hepatocellular carcinomas cell subtypes but is repressed in “poorly differentiated” subtypes [73, 77]. Also, potentiated canonical Wnt signaling may contribute to glioblastoma cell growth through maintaining cancer stemness trait and stimulating cancer metastasis [75]. In contrast, activation of non-canonical Wnt signaling has been demonstrated to inhibit tumor growth [73, 74, 78], possibly mediated by antagonizing canonical Wnt signaling [73].

## Lgr5 in malignant hematopoiesis

Lgr5, a Wnt target gene, has been widely used as a marker of organ stem cells with self-renewal capacity [41, 79], as well as an established biomarker of cancer stem cells (e.g., colorectal cancer and mammary tumors) [80]. Simultaneously, Lgr5 has been recently reported to be essential for B cell development. Self-renewal in the B cell lineage is induced by positive selection and antigen-receptor (BCR) signaling, i.e., an encounter of cognate antigen. Self-renewal at this stage leads to clonal expansion and survival. Studies have demonstrated Lgr5 expression in B cells, which restricts the levels of nuclear β-catenin and enables B cell survival through negative regulation of canonical Wnt-signaling, although most studies suggest the potentiated role of Lgr5 in the regulation of Wnt signaling [81]. Thus, Lgr5 enables positive B cell selection and tumor initiation in B cell malignancies [82]. Lgr5 may become a potential diagnostic marker and therapeutic target for B cell malignancies.

## Targeting Lgr5^+^ cancer stem cells

Cancer stem cells (CSCs) have been identified as a driving force in tumor initiation, growth, and metastases [83, 84]. Also, CSCs are believed to play central roles in drug resistance and resistance of standard chemotherapy and radiation treatment [85–88]. Cancer cells in primary solid tumors reside in a complex microenvironment comprising numerous cell types, including endothelial cells of the blood vessels, lymphatics, pericytes, adipocytes, and various bone marrow-derived cells, such as macrophages, platelets, and mesenchymal stem cells [89–91].

Despite stem cell niches of different tissues and species share many structural similarities, they differ in their functional characteristics [92]. Many CSC biomarkers are emerging as prognostic factors for carcinogenesis, tumor aggressiveness and, therapeutic resistance. Studies have demonstrated that CSCs could be characterized using markers, such as CD133 [83, 93], CD24/29 [94, 95], or a combination of CD44 and CD166 [96]. Strikingly, Lgr5 has been referred to as a novel biomarker of various human cancers of the stomach [97], pancreas [98, 99], liver [100], colon [101], and ovary [102] during recent years. Notably, CSCs are critical for the formation and maintenance of tumor growth and metastasis, which may represent a therapeutic opportunity for cancer therapy. However, until now, whether a single CSC-targeting therapy is sufficient to eradicate cancers still remains controversial, considering the potential treatment evasion by non-CSC plasticity [103].

Enhanced Wnt signaling was detected in CSCs due to the elevated β-catenin localization within the nuclear region. Interestingly, Lgr5, a cell surface-expressed Wnt target gene, has been demonstrated as a functional biomarker of cancer stem cells, contributing to cancer stem cell proliferation and self-renewal through the regulation of Wnt/β-catenin signaling pathway. Notably, the expression of Lgr5 is reported to be increased in human colorectal adenomas and cancers [104–106], hepatocellular carcinoma, basal cell carcinoma, and neuroblastoma [55, 107, 108]. Lgr5^+^ cells have been demonstrated to be the cells of origin of tumors [109], which may provide a feasible approach for effective targeted anti-tumor treatment through targeted elimination of Lgr5^+^ CSCs selectively.

A recent study reported that targeting Lgr5^+^ cells with an antibody conjugated to distinct drugs exhibited potent efficacy to decrease tumor size and proliferation of colon cancer [110]. Simultaneously, a similar study further confirmed the tumor eradicative and recurrence-preventive effects through Lgr5-targeted antibody-drug conjugates in a xenograft model of colon cancer [111]. Furthermore, Lgr5^+^ CSC-targeted drug delivery system might also become a promising approach for targeted anti-tumor therapy. A recent study reported that RSPO1 (a Lgr5 natural ligand)-conjugated liposomes encapsulating doxorubicin led to massive tumor tissue necrosis and growth inhibition through efficient targeting of Lgr5^+^ CSCs in a patient-derived xenograft tumor model [112]. However, studies have also shown that selective ablation of Lgr5^+^ CSCs using gene-specific approach is effective temporarily yet is eventually overcome by robust plasticity of non-targeted cancer cells [65]. Hence, the reappearance of Lgr5^+^ cells following complete elimination of the Lgr5^+^ colorectal cancers may result from the plasticity of Lgr5^−^ colorectal cancers and the transition between Lgr5^+^ CSCs and Lgr5^−^ CSCs [66]. Hence, accumulating preclinical evidence have suggested the potential of targeting Lgr5 via Lgr5-targeted antibody-drug conjugates as effective novel therapeutics for tumor treatment [110, 111, 113]. A single targeting of Lgr5^+^ cancer cells may not be able to eradicate cancers and prevent tumor recurrence sufficiently for some certain cancer types at a specific stage. Furthermore, a combined therapy targeting both Lgr5^+^ and Lgr5^−^ cancer cells may desire further consideration and experimental validation.

In addition, drug screening targeting Lgr5-associated signaling pathways in tumor organoids *ex vivo* might provide a powerful tool for the development of novel drugs for personalized anti-tumor therapy [114–119]. Also, Lgr5, a surface-expressed protein, may become a potential therapeutic target for tumor therapy through antibody-based or drug delivery therapies. Simultaneously, more importantly, more comprehensive studies on the plasticity of cancer cells within a solid tumor are warranted to the recurrence of Lgr5^+^ cells following targeted elimination of Lgr5^+^ cancer cells, which may be instrumental for the development of novel strategies for more effective anti-tumor treatments.

## Multifaceted roles of Lgr5 during cancer progression

Generally, numerous reports have indicated that Lgr5 promotes both development and survival of various cancer types through potentiation of Wnt/β-catenin signaling, such as colorectal cancer, and glioblastomas [120]. Also, studies have demonstrated close associations between Lgr5 and aggressiveness in neuroblastoma cell lines at different stages of treatment [121], as well as in papillary thyroid cancer [122]. Further studies have indicated the involvement of Lgr5 in drug resistance through Wnt signaling in neuroblastoma cell lines [120]. Therefore, increasing evidence suggest that targeted suppression of Lgr5 in some certain cancer types may represent potential therapeutic approaches for treating cancer.

**Fig. 2:**
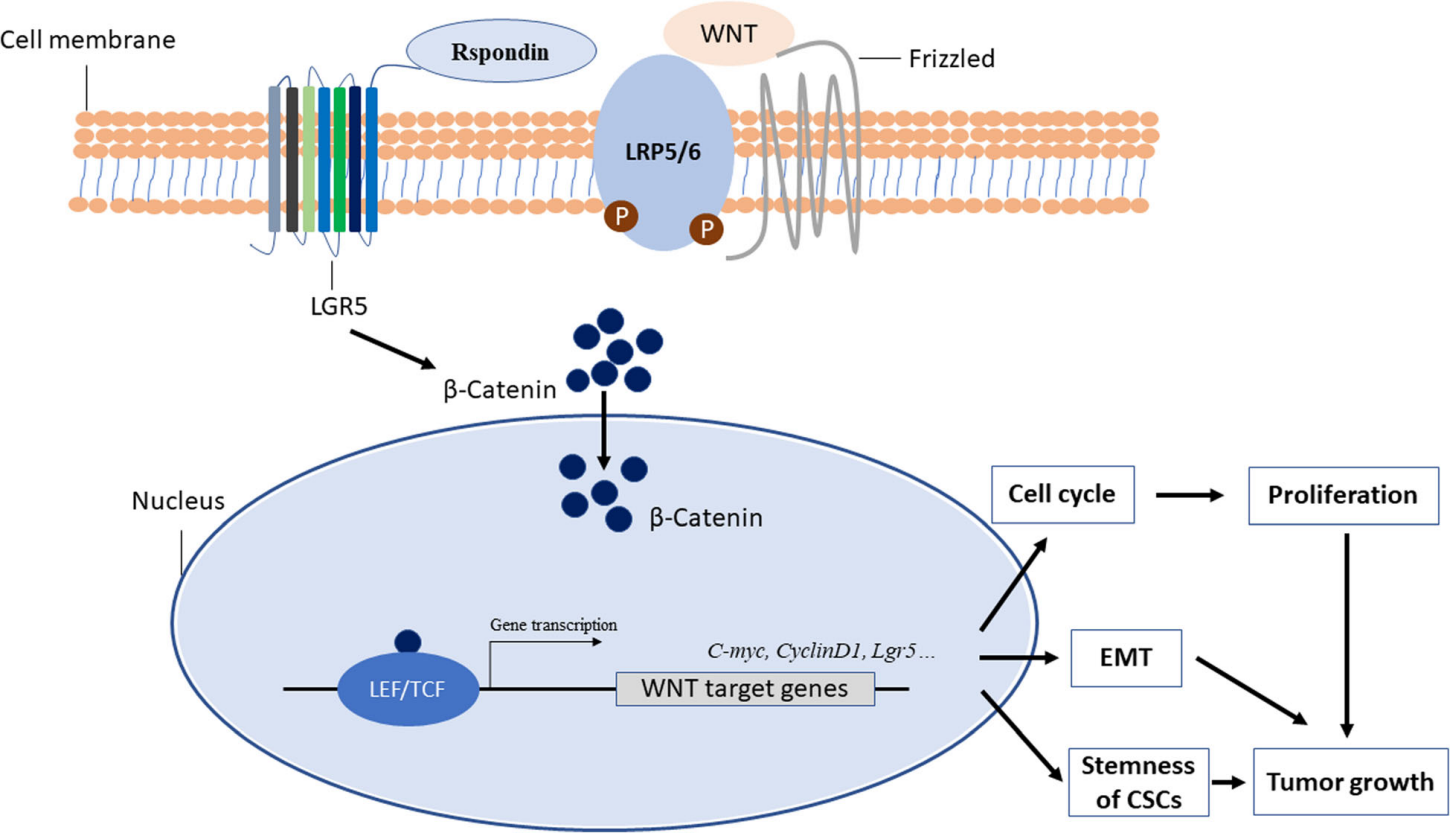
Proposed model of the Lgr5-mediated stimulation of cancer growth through activation of the Wnt/β-catenin signaling pathway. R-spondin activates Leucine-rich repeat-containing G protein-coupled receptor 5 (Lgr5). Once activated, Lgr5 protein recruits LRP-frizzled receptor complex, which binds to Wnt ligands, reinforcing Wnt signaling following phosphorylation of LRP5/6. A series of steps ensue, including the accumulation of β-catenin, which is translocated to the nucleus, inducing the expression of various Wnt target genes (such as C-myc, Cyclin D1, Lgr5) after binding together with the TCF/LEF family of transcription factors, which leads to tumor progression through stimulation of cell proliferation, epithelial-mesenchymal-transition (EMT), and stemness maintenance of cancer stem cells (CSCs). TCF, T cell-factor; LEF, lymphoid enhancer-binding factor

However, until now, the prognostic significance of Lgr5 in cancer still remains controversial. Lgr5 may exert multiple or even different functions between different cancer types. Discrepant results of reports on the effects of Lgr5 expression in tumor progression have been presented in the literature. Reports of Lgr5-mediated tumor growth in human glioma have been listed in Table 1, whilst studies on Lgr5-induced tumor suppression in human colorectal cancer listed in Table 2. Numerous studies have reported the stimulatory effects of Lgr5 in tumor growth through the regulation of CSC stemness, epithelial-mesenchymal transition (EMT), and cancer cell proliferation (Fig. 2) [58, 108, 123, 127].

**Table 1:** Lgr5-mediated stimulation of tumor growth in glioma

Authors	Tumor models	Conclusions	Mechanisms
Vieira et al. [108]	Neuroblastoma cell lines (SK-N-BE(2)-C, IMR32, NGP, SH-SY5Y, GIMEN, and SK-N-NAS)	Lgr5 stimulated tumor growth and proliferation	Regulation of MEK/ERK and Akt pro-survival signaling pathways
Zhang et al. [123]	Adult glioma patients; primary glioma cells	Lgr5 is a new functional glioma stem cell (GSC) marker involved in EMT and prognostic indicator of glioma	Stimulation of EMT by activating the Wnt/β-catenin pathway and maintenance of GSC stemness by modulating the expression of SOX2, Nanog, CD133, CD44, CD24, and EpCAM
Xie et al. [60]	Glioblastoma patient-derived stem cell (GSC) cultures	Stimulation of tumorigenicity and invasion by Lgr5	Regulation of the interactions between tumor cells and their microenvironment through extracellular matrix and collagen signaling pathways

**Table 2:** Lgr5-mediated tumor suppression in colorectal cancer

Authors	Tumor models	Conclusions	Mechanisms
Jang et al. [124]	Colorectal cancer (CRC) samples collected from patients	Lgr5 functions as a tumor suppressor in the late stages of CRC progression	Attenuation of proliferation, migration, and colony formation of colon cancer cells
Zhou et al. [125]	Human colon carcinoma cell lines (HCT116, RKO, FET, CBS, HCT116b, and TENN)	Lgr5 inhibited cell survival and clonogenic of CRC in vitro; suppression of metastasis of CRC in vivo	R-spondin 1/Lgr5-induced activation of TGF-β signaling with TGF-β type II receptor, inducing inhibition and apoptosis of CRC
Wu [126]	Primary colorectal tumors from CRC patients; CRC cell lines (HEK293, LS174T, DLD1, HT29, and HCT116)	Inhibition of tumor growth of CRC	RSPO2-induced, Lgr5-dependent Wnt signaling-negative feedback loop

Studies have shown that Lgr5 is implicated in tumor growth and metastasis via various signaling pathways. For example, during the progression of neuroblastoma development, Lgr5 enhanced tumor survival and proliferation through the regulation of phosphorylation of mitogen/extracellular signal-regulated kinases (MEK1/2) and extracellular signal-regulated kinases (ERK1/2), besides through reinforcement of Wnt/β-catenin signaling [108]. Additionally, nuclear IκB-kinase α (IKKα) could directly bind to the promoters of inflammation factors and LGR5, which upregulates Lgr5 expression in turn through the activation of STAT3 signaling pathway during cancer progression of basal cell carcinomas [128]. It is generally believed that the activation of nuclear factor (NF)-κB inflammatory pathway leads to a pro-tumorigenic inflammatory microenvironment [129, 130]. And the IκB-kinase complex (IKKα and IKKβ) and its regulatory subunit (IKKγ) regulate the NF-κB signaling [129, 131]. Thus, NF-κB signaling-induced abnormal immune response during cancer development may be another important stimulating factor of Lgr5 expression.

In contrast, a recent study has reported a suppressive role of Lgr5 during the late stages of colorectal cancer progression through attenuation of growth, colony formation, and migration capacities of colon cancer cells [124]. Also, Lgr5 is known to exhibit tumor-suppressive activity in the colon, which results from suppression of tumor growth and metastasis regulated by RSPO1/Lgr5-mediated activation of TGFβ signaling [125]. Furthermore, studies have reported the RSPO2-induced, Lgr5-dependent Wnt signaling-negative feedback loop, exhibiting a tumor-suppressive activity in colorectal tumors [126]. Therefore, Lgr5 may exert complicated or even opposing functions during the progression of different cancer types.

## Potential crosstalk between Lgr5^+^ stem cells and immune cells

Inflammation has been shown as a hallmark of cancer development [132]. Chronic inflammation, which is caused by exposure to environmental agents, infection, genetic diseases, and metabolic disorders, is tightly correlated with various tumors, including lung carcinoma, gastric cancer, cervical cancer, colorectal cancer, hepatocellular carcinoma, and multiple myelomas [133, 134].

Resident immune cell populations of the mammalian innate immune system mainly comprise of macrophages, dendritic cells, and adaptive immune T cells [135]. In particular, Tregs appear to be important regulators of adult stem cells under steady-state conditions [136]. Interestingly, Tregs have been demonstrated to preserve the integrity of Lgr5^+^ intestinal stem cells in the intestine, whilst depletion of Tregs would cause a dramatic reduction in the number of intestinal stem cells [137]. Also, co-culture of intestinal organoid cultures with Tregs or their effector cytokine IL-10 induced a rapid enrichment of Lgr5^+^ intestinal stem cell pool significantly *ex vivo* [137]. Therefore, interactions between T helper cells and Lgr5^+^ stem cells may probably take place during the progression of tumor-promoting immune microenvironment of chronic inflammation due to their proximity, which may play critical roles in tumor growth and metastasis.

## Safety concerns over Lgr5-targeted therapy

Although previous studies have shown some therapeutic effects of Lgr5-targeted anti-cancer therapy, there still exist some safety issues due to the presence of Lgr5-expressing adult stem cells in adult mammals [138–141]. Hence, considering the expression of Lgr5 in various normal adult tissues as a bona fide marker of adult stem cells, a more specific targeting using double biomarkers (such as Lgr5 and CD133) may exhibit much safer and tissue-specific targeting efficacy. In the meanwhile, targeted modulation of Lgr5 expression in solid tumors through targeted genome editing system or specific drug delivery system carrying natural compounds, engineered nanoparticles, or Chinese herb screened might become promising approaches and important research directions for targeted anti-cancer therapy.

## Conclusion

Taken together, Lgr5 emerges as a potential surface-expressing biomarker for targeted anti-tumor therapy. Lgr5 is crucial for tumor initiation, progression, and metastasis through regulation of CSC function, Wnt/β-catenin signaling pathway, and various other signaling pathways. Lgr5 plays complicated and multifaceted roles during tumor progression. Lgr5-associated signaling pathways may play different or even opposing roles in different cancer types. Thus, targeted therapeutic modulation of Lgr5-associated signaling pathways may provide potential opportunities for anti-cancer therapy.

Single targeting Lgr5^+^ CSCs in solid cancer through antibody-conjugated drug delivery system exhibited some anti-cancer therapeutic effects. However, although selective targeting of Lgr5^+^ CSCs through genetic ablation is effective to treat tumor temporarily, the anti-tumor effects would be overcome by non-targeted cancer cells eventually. Thus, a combined therapy targeting both Lgr5^+^ and Lgr5^−^ cancer populations may deserve further consideration. Further comprehensive studies investigating the plasticity of cancer cells and transitions between Lgr5^−^ and Lgr5^+^ cancer cells are necessary. Drug screening for *ex vivo* tumor organoids may be promising for the development of personalized drugs treating tumor. Meanwhile, a more specific targeting of Lgr5-expressing tumor cells using double biomarkers may deserve further consideration and elucidation.

## Data Availability

Please contact the author for data requests.

## Abbreviations

CRC: Colorectal cancer
CSCs: Cancer stem cells
EMT: Epithelial-mesenchymal transition
GPCR: G protein-coupled receptor
GPR49: G protein-coupled receptor 49
GSC: Glioma stem cell
IL-33: Interleukin-33
Lgr5: Leucine-rich repeat-containing G protein-coupled receptor 5
SDF-1: Stromal cell-derived factor 1
TNF-α: Tumor necrosis factor-α
Tregs: Regulatory T cells
